# Commencement

**Published:** 1878-04

**Authors:** 


### COMMENCEMENT.

The third annual commencement of the Dental College of the University of Michigan was held on Wednesday, March 27th, in the hall of the University, in the presence of a very large audience. The commencement exercises of the Medical College were held at the same time.

The candidates for graduation from the Dental Department were as follows, viz.

Edwin Earle Arnold, Mich.; John Norman Cowan, Mich.; Thomas Sears Ewing, Mich.; Sherman Fremont Finch, Mich.; Mark Florus Finley, Mich.; Alvin Knapp, Ind.; Fremont Ernest Lyon, Ohio; James Buchanan McGregor, Mich.; Benjamin Franklin Miller, Mich.; William H. Miller, Ohio; William Mitchell, Ohio; Eben Levi Parker, Mich.; Charles Warren Sanderson, N. Y.; George Henry Wilson, Ohio.

The annual address was delivered by Dr. Geo. L. Field, of Detroit.

